# Pediatric Jugular Bulb Anomalies: Management Insights From Two Cases

**DOI:** 10.7759/cureus.114187

**Published:** 2026-08-09

**Authors:** Shawky Francis, Raanan Cohen-Kerem

**Affiliations:** 1 Ear, Nose, Throat (ENT), Carmel Medical Center, Haifa, ISR; 2 Otolaryngology - Head and Neck Surgery, Carmel Medical Center, Haifa, ISR

**Keywords:** conductive hearing loss, hemorrhage, high riding jugular bulb, jugular bulb anomalies, tympanostomy tube

## Abstract

Jugular bulb anomalies are rare but important causes of pediatric conductive hearing loss. These variants can disrupt ossicular mechanics or alter middle ear function and may persist despite tympanostomy tube placement. We report two cases: a five-year-old boy with unilateral conductive hearing loss due to high-riding bulb, managed conservatively, and an eight-year-old boy with persistent conductive hearing loss post-tympanostomy, in whom intraoperative hemorrhage occurred during middle ear exploration due to jugular bulb dehiscence.

## Introduction

Jugular bulb anomalies, though uncommon and often overlooked, are increasingly recognized as potential causes of conductive hearing loss (CHL) in children. Anatomical variations such as high-riding jugular bulb (HRJB) or jugular bulb dehiscence (JBD) may exert pressure on the surrounding structures, particularly the middle ear, disrupting auditory function. This impairment typically arises from either interference with the ossicular chain or modifications of middle ear dynamics [1]. While tympanostomy tube insertion remains a standard and widely used treatment for pediatric CHL, certain cases demonstrate persistent symptoms due to underlying jugular bulb anomalies [2]. Furthermore, a high jugular bulb presents notable surgical challenges, underscoring the importance of comprehensive preoperative assessment to mitigate intraoperative risks.

Several studies have highlighted the uncommon yet clinically significant link between jugular bulb anomalies and CHL. Young Hoon Koo et al. reported cases in which JBD was identified as the primary source of CHL, reinforcing the idea that these vascular anomalies may closely mimic more frequently encountered otologic conditions [3]. In many cases where CHL results from mechanical interference by the jugular bulb with sound transmission through the middle ear, surgical treatment often fails to yield measurable hearing improvement [2,4].

We report two cases of jugular bulb anomalies. The first case involves a five-year-old boy with persistent unilateral CHL caused by HRJB, for which a conservative approach was chosen. The second case describes an eight-year-old boy with persistent CHL, even after tympanostomy tube placement, who underwent elective tympanoplasty because of adhesive otitis media. During the procedure, a significant hemorrhage occurred when the tympanomeatal flap was raised. We describe and discuss the management strategy and treatment options here.

This article was previously posted to the Authorea preprint server on September 25, 2025 [5].

## Case presentation

Case 1

**Figure 1:**
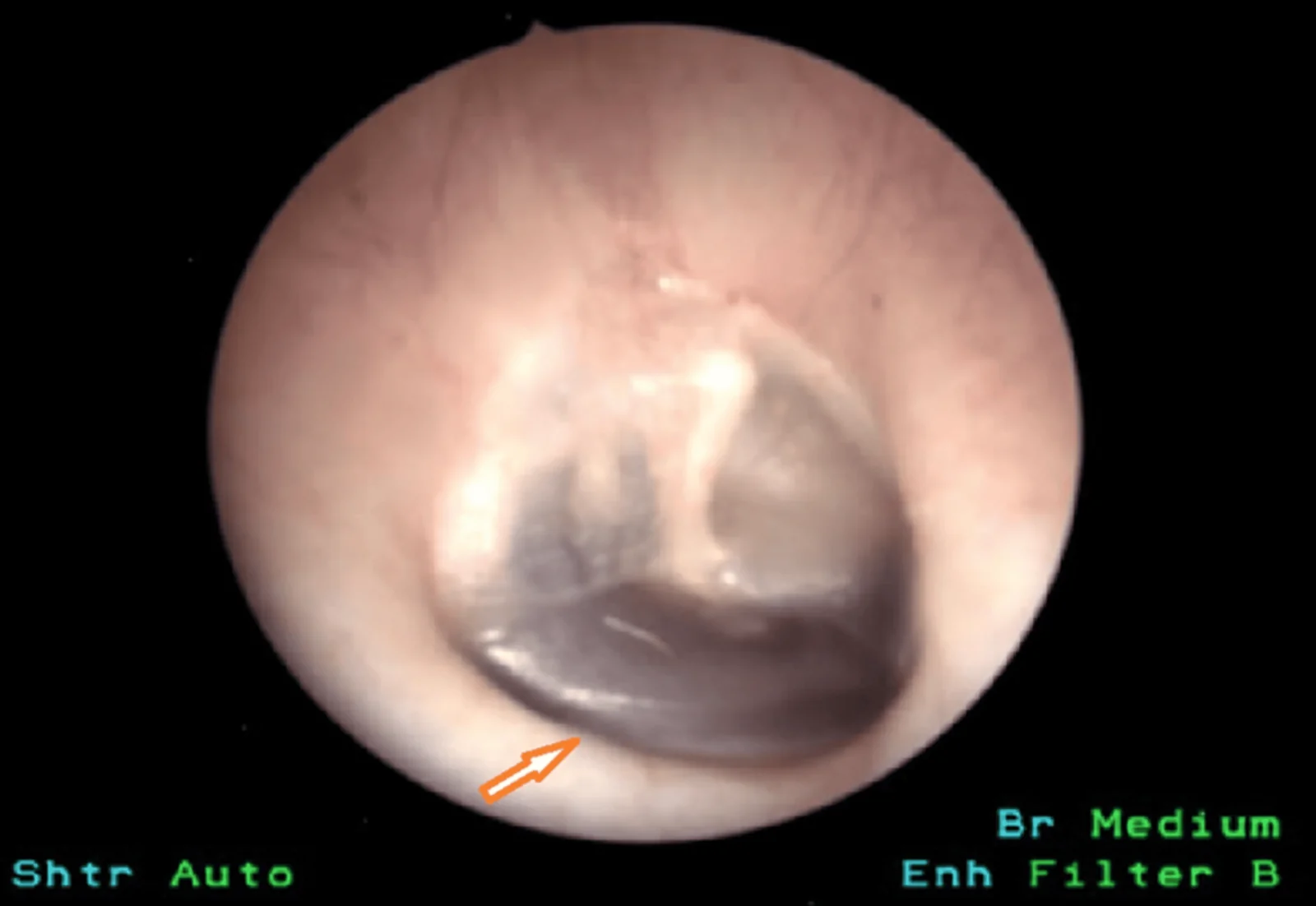
High-riding jugular bulb in the right ear during endoscopy. Arrow indicating the jugular blub.

**Figure 2:**
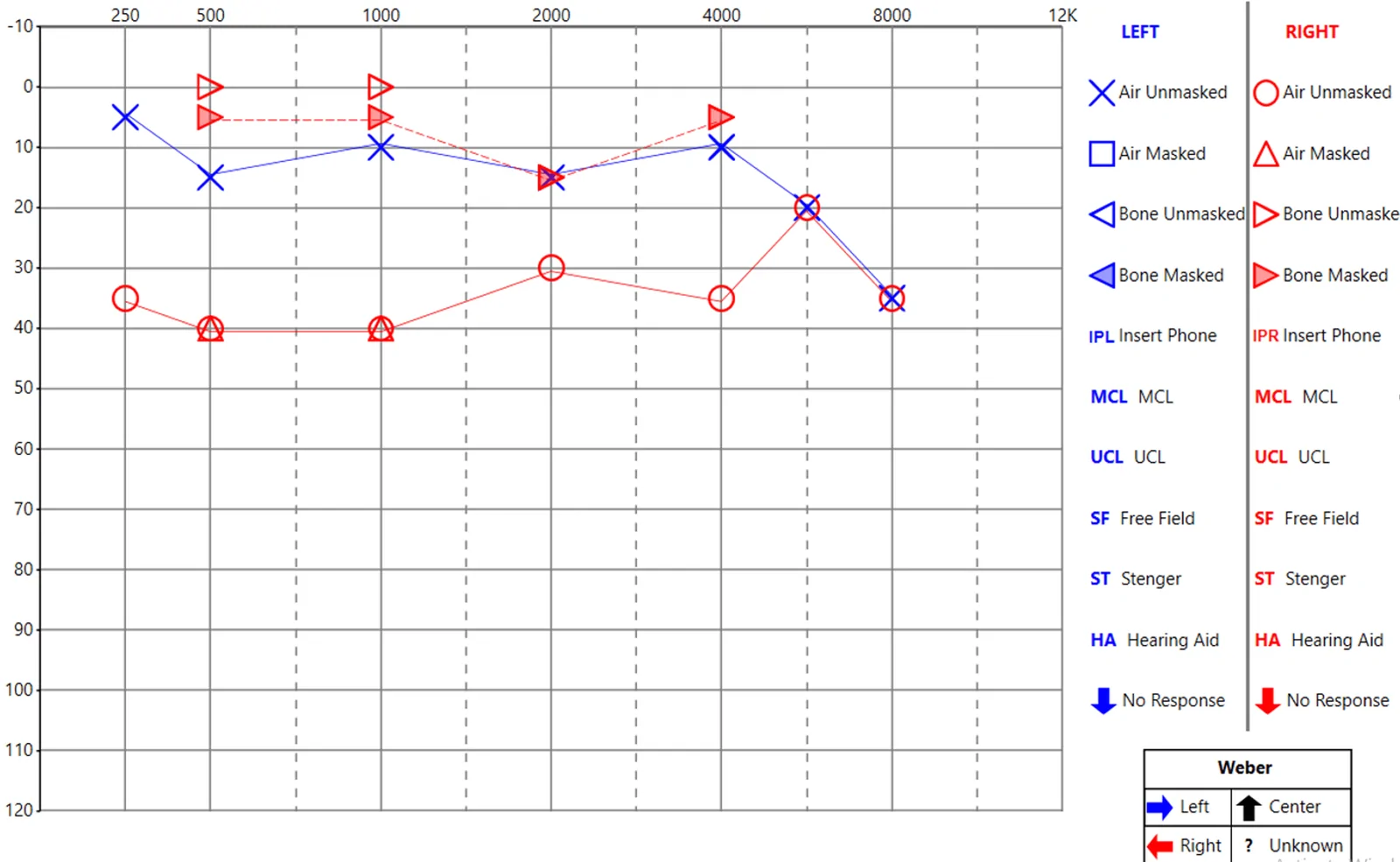
Audiometry demonstrated conductive hearing loss of 30-40 dB across all frequencies

**Figure 3:**
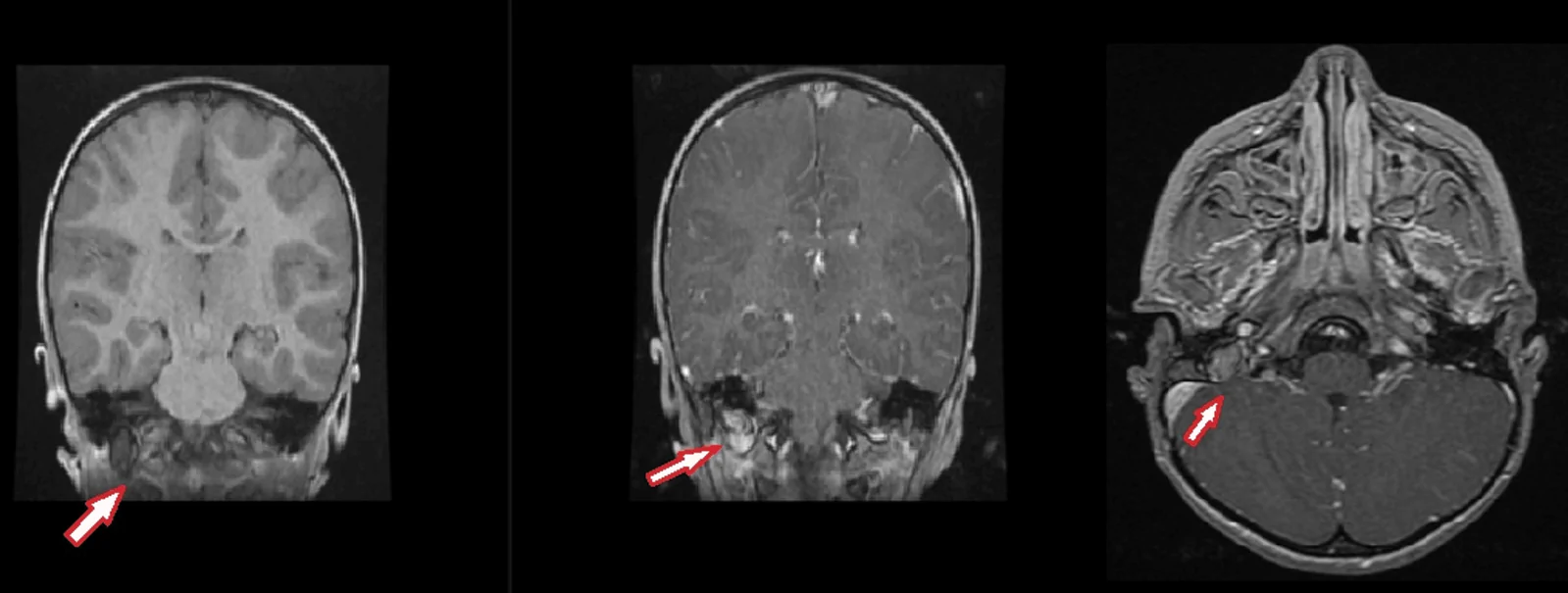
MRI of the temporal bone showing a protrusion of the jugular bulb into the right middle ear, consistent with a HRJB (arrows). Left image: Coronal section from T1-weighted MRI without contrast showing hypointense JB (indicated by the arrow). Middle image: Coronal section from T1-weighted MRI with fat saturation and IV contrast showing prominent enhancement of JB (indicated by the arrow). Right image: Axial section from T1-weighted MRI with fat saturation and IV contrast showing prominent enhancement of JB (indicated by the arrow). HRJB: high-riding jugular bulb; JB: jugular bulb.

A five-year-old boy, healthy, born at 30 weeks’ gestation, was referred to our institution for evaluation of right sided unilateral CHL. Otoscopic examination revealed a bluish discoloration posterior to the right tympanic membrane (Figure 1). Audiometry demonstrated conductive hearing loss of 30 to 40 dB across all frequencies in the right ear, with normal bone conduction threshold. The left ear exhibited normal hearing (Figure 2). MRI of the temporal bone showed a protrusion of the jugular bulb into the right middle ear, consistent with a HRJB (Figure 3). Given the moderate degree of hearing loss and the benign nature of the HRJB, a conservative management strategy was implemented. The patient was fitted with a hearing aid in the affected ear, which significantly improved his hearing. Ongoing follow-up is planned to monitor auditory function.

Case 2

**Figure 4:**
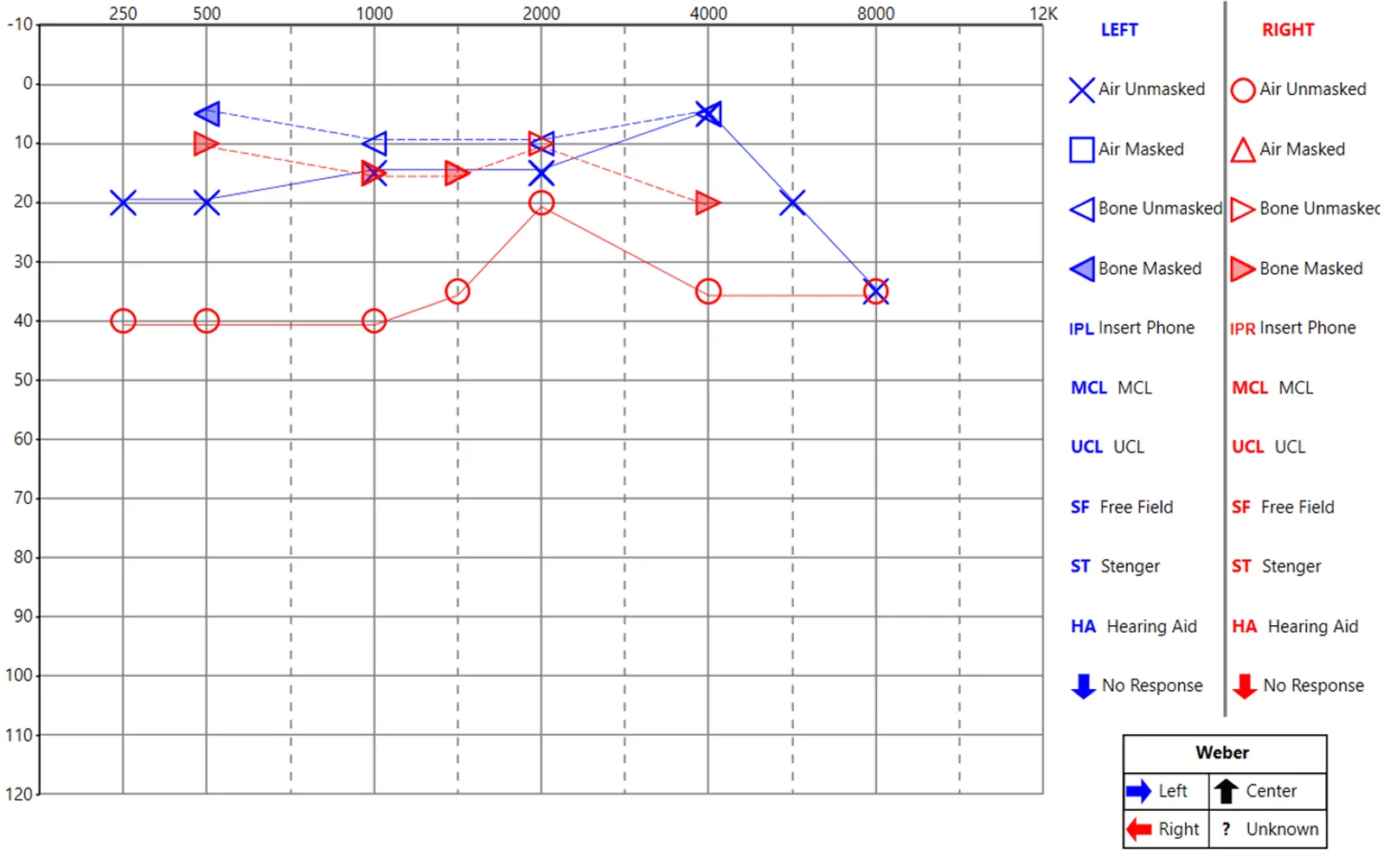
Audiometry revealing normal hearing in the left ear, but CHL in the right ear with an SRT of 40 dB and type B tympanogram

**Figure 5:**
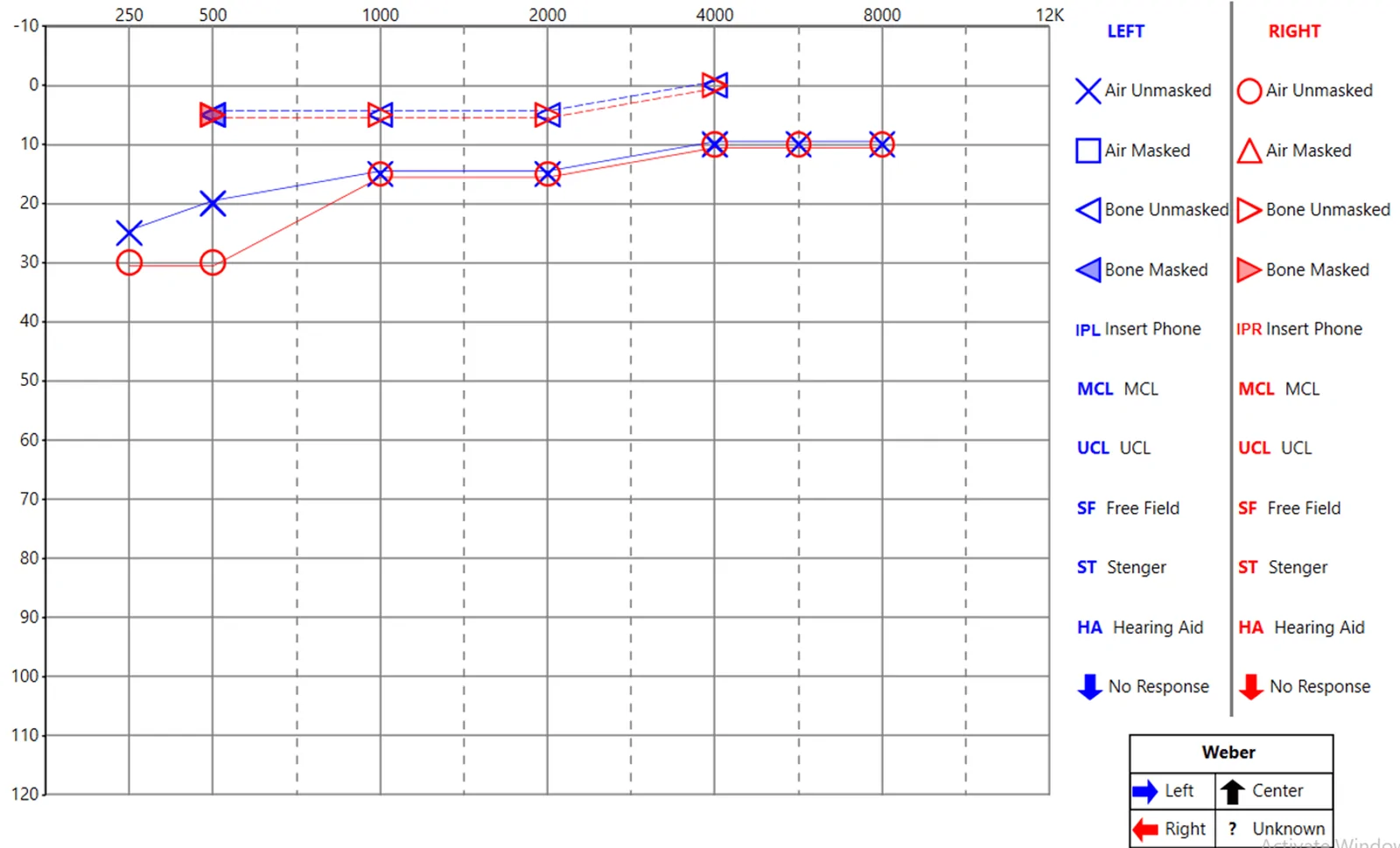
Follow-up hearing test demonstrated symmetric hearing threshold of 20 dB bilaterally, with and a conductive gap of 10 dB in both ears

An otherwise healthy eight-year-old boy underwent bilateral middle ear ventilation tube insertion at the age of four due to bilateral CHL, resulting in improved hearing thresholds of 10 dB in both ears. Subsequently, his parents observed a decline in hearing. At age six, a hearing test (Figure 4) revealed normal hearing in the left ear, but CHL in the right ear with a speech recognition threshold (SRT) of 40 dB and type B tympanogram. A repeat ventilation tube placement in the right ear was attempted but unsuccessful due to extensive tympanic membrane adhesion and a markedly small tympanic cavity. At age seven, a follow-up hearing test (Figure 5) demonstrated a symmetric hearing threshold of 20 dB bilaterally, with a conductive gap of 10 dB in both ears. He was referred for adenoidectomy; however, no significant improvement in hearing thresholds was observed postoperatively. Subsequent audiometry indicated the following findings: Left ear - minimal low-frequency CHL with an air-bone gap of 15 dB, and mild high-frequency sensorineural hearing loss at a threshold of 15 dB. Tympanometry remained type B. Right ear - mild hearing loss across all frequencies, with an air-bone gap of 30 dB, and noted improvement at 2000 Hz. SRT was measured at 35 dB, with type B tympanometry.

Based on the audiological and clinical findings, the patient was referred for trans-canal endoscopic exploration of the right ear.

**Figure 6:**
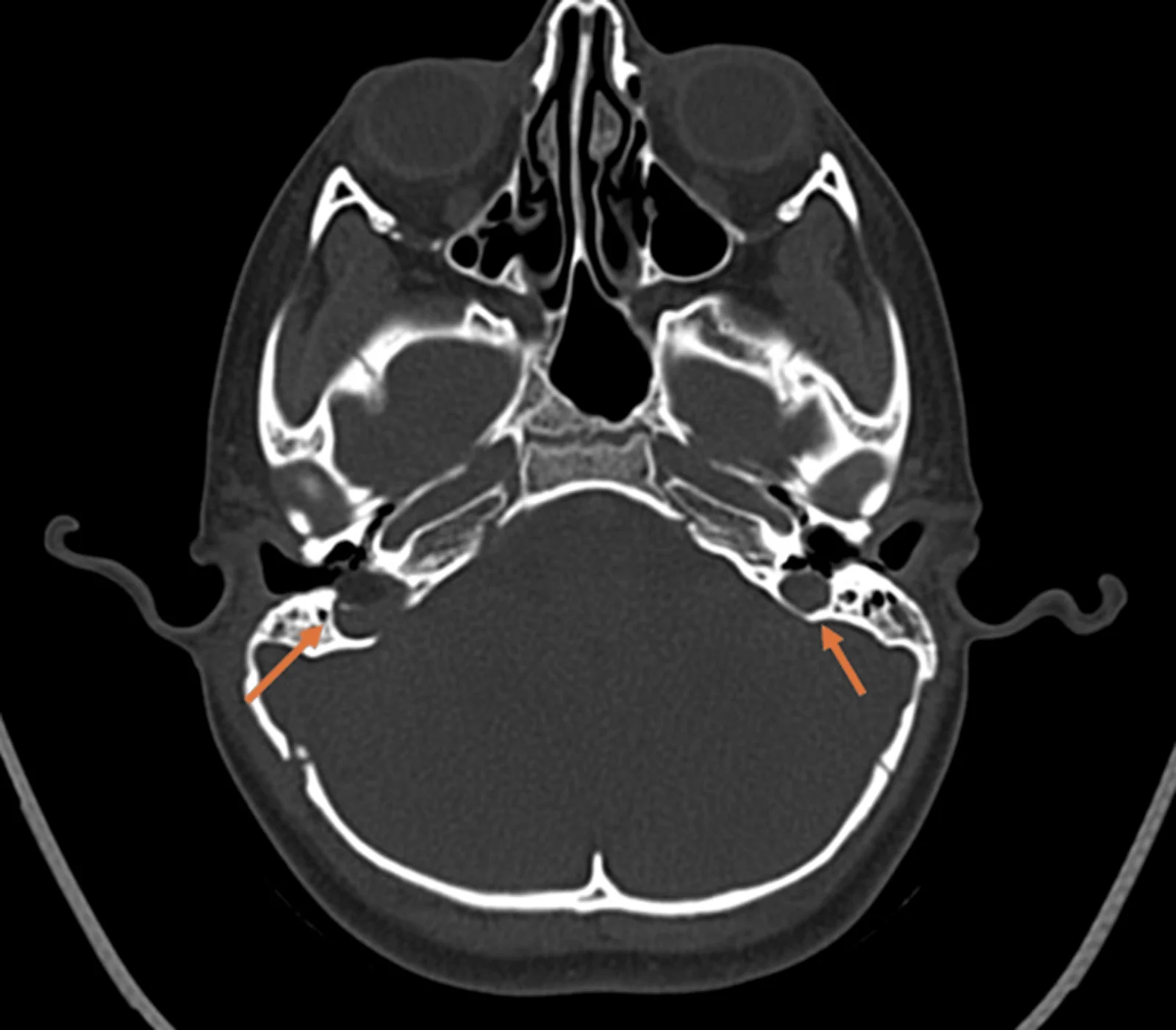
Axial section of CT scan of the temporal bones without contrast, revealing bilateral DJB covered by thin bony plate. (arrows on both sides are indicating the DJB). DJB: Dehiscent jugular bulb.

Preoperative radiological assessment was deferred, as the clinical presentation was highly suggestive of standard middle ear pathology, such as ossicular chain fixation or discontinuity. Furthermore, given the patient’s young age, the surgical team elected to avoid unnecessary radiation exposure, reserving computed tomography (CT) imaging for potential future indications. Intraoperatively, the tympanic membrane appeared opaque and atelectatic. It was adhered inferiorly to the promontory, and superiorly to the malleus handle, lenticular process, and incudo-stapedial joint. During elevation of the tympanomeatal flap, at approximately the 6 o'clock position of the annulus, brisk bleeding was encountered. Multiple attempts to localize the source were unsuccessful. Hemostasis was eventually achieved through packing and the decision was made to abort the procedure and plan for re-exploration at a later date. The patient’s hemoglobin level dropped from 11 g/dL to 7.5 g/dL. A subsequent CT scan of the temporal bones revealed bilateral dehiscent jugular bulb (DJB) covered by thin bony plate (Figure 6). Three days later, during the second-look procedure via a trans canal approach, removal of the packing led to renewed brisk hemorrhage. After several unsuccessful attempts to control the bleeding, a retro-auricular incision was performed to obtain improved access. The bleeding source - a defect over the jugular bulb - was successfully sealed with bone wax, achieving definitive hemostasis.

During hospitalization, the patient developed facial nerve palsy, which was managed conservatively. Full recovery was observed within five weeks. At present, the tympanic membrane remains perforated and repair is planned in the near future.

## Discussion

Jugular bulb anomalies represent vascular variations arising from developmental alterations in the jugular vein's position and prominence. The main subtypes include HRJB, DJB, and aberrant jugular bulb. Although well described in adults, its presentation in children is rare, making these cases noteworthy [6].

In both pediatric cases, the jugular bulb anomaly manifested primarily as CHL. The first case presented with a bluish retro-tympanic mass visible on otoscopy. The second case was first suspected during middle ear exploration when unexpected intraoperative bleeding occurred, revealing the vascular anomaly. These findings underscore how JBA may mimic other middle ear masses or adhesions.

CHL in jugular bulb anomalies can result from: (1) middle ear invasion - a dehiscent or high-riding jugular bulb may protrude into the middle ear cavity, restricting ossicular chain mobility and disrupting sound transmission [1]; (2) bone erosion - chronic pressure from an enlarged jugular bulb can cause sigmoid plate resorption, exposing vascular tissue and elevating the risk of intraoperative bleeding [7]; and (3) tympanic membrane alterations - persistent negative middle ear pressure or adhesions (as seen in Case 2) may induce tympanic membrane retraction and middle ear remodeling, further worsening CHL [3].

Accurate diagnosis and differentiation of JBA from other retrotympanic lesions (e.g., glomus tumors, aberrant carotid arteries, cholesterol granulomas) rely on imaging [8]. High-resolution CT of the temporal bone reveals dehiscence of the sigmoid plate and quantifies the jugular bulb's extension into the middle ear space [9]. Magnetic resonance imaging differentiates vascular anomalies from soft tissue masses and evaluates venous involvement [10]. When unexpected brisk bleeding occurs intraoperatively - suggesting an unrecognized vascular anomaly in the absence of preoperative imaging - the surgeon should immediately achieve hemostasis, halt further dissection and obtain targeted imaging as soon as feasible to guide safe continuation of the procedure.

High and dehiscent jugular bulbs pose significant intraoperative hazards in ear surgery. Regarding prevalence and risk, approximately 2% of patients fall into a ‘‘high-risk’’ category for jugular bulb variants, with a right ear and male predominance [11]. Preoperative assessment using CT angiography and venography should be obtained when JBA is suspected to map vascular anatomy and minimize bleeding risks [12]. Intraoperative hemorrhage control utilizing an exclusive endoscopic approach can effectively manage bulb bleeding without conversion to a microscopic approach [13].

Treatment must be individualized based on symptom severity and hearing impairment. In the first case, the presence of HRJB was noted clinically and radiologically but did not require immediate intervention beyond amplification and periodic audiometric monitoring. However, in the second case, an attempted middle ear exploration led to significant bleeding, likely due to JBD or a vascularized adhesion to the tympanic membrane. This highlights the need for preoperative suspicion to anticipate vascular anomalies, avoiding aggressive manipulation in cases where a vascular mass is suspected, considering alternate strategies.

Management of JBA is typically conservative, with surgical intervention reserved for cases with significant symptoms or progressive hearing loss. Long-term follow-up should include audiometric monitoring, repeat imaging as needed, counselling families on symptoms such as tinnitus or dizziness, which may indicate further vascular involvement.

## Conclusions

These cases highlight the need to recognize jugular bulb anomalies as an uncommon but important cause of pediatric CHL. Early radiologic assessment, meticulous surgical planning, and a multidisciplinary strategy involving otologists and radiologists are essential for safe, effective management. Because intraoperative hemorrhage can be catastrophic, surgeons should maintain a high index of suspicion for vascular anomalies whenever they encounter unexplained tympanic mass or unexpected bleeding. In the event of brisk intraoperative hemorrhage, converting to a retro-auricular approach may be necessary to gain optimal vascular control.
